# How A Patient with Resectable or Borderline Resectable Pancreatic Cancer should Be Treated—A Comprehensive Review

**DOI:** 10.3390/cancers15174275

**Published:** 2023-08-26

**Authors:** Daria Kwaśniewska, Marta Fudalej, Paweł Nurzyński, Anna Badowska-Kozakiewicz, Aleksandra Czerw, Elżbieta Cipora, Katarzyna Sygit, Ewa Bandurska, Andrzej Deptała

**Affiliations:** 1 Department of Oncology, Central Clinical Hospital of the Ministry of Interior and Administration, 02-507 Warsaw, Poland; kwasniewska@post.pl (D.K.); marta.fudalej@wum.edu.pl (M.F.);; 2 Department of Oncology Propaedeutics, Medical University of Warsaw, 01-445 Warsaw, Poland; 3 Department of Health Economics and Medical Law, Medical University of Warsaw, 01-445 Warsaw, Poland; 4 Department of Economic and System Analyses, National Institute of Public Health NIH-National Research Institute, 00-791 Warsaw, Poland; 5 Medical Institute, Jan Grodek State University in Sanok, 38-500 Sanok, Poland; 6 Faculty of Health Sciences, Calisia University, 62-800 Kalisz, Poland; 7 Centre for Competence Development, Integrated Care and e-Health, Medical University of Gdansk, 80-204 Gdansk, Poland

**Keywords:** pancreatic cancer, resectable, borderline resectable, adjuvant, neoadjuvant, resection, chemotherapy, radiotherapy

## Abstract

**Simple Summary:**

Recently observed improvement in the treatment of pancreatic ductal adenocarcinoma (PDAC) has resulted mainly from improved perioperative care and more effective chemotherapy. Over the past decade, there has been a gradual increase in the number of treatment options for pancreatic conditions. The PRODIGE 24 trial established mFOLFIRINOX as the standard of care in adjuvant therapy, demonstrating a significant benefit in terms of overall survival. Questions remain as to how to optimize neoadjuvant chemotherapy and who would benefit from upfront surgery instead of neoadjuvant chemotherapy. In addition, the role of chemoradiotherapy is not clearly established. The article presents our treatment plan for early pancreatic cancer supported by current research results.

**Abstract:**

Pancreatic ductal adenocarcinoma (PDAC) is an aggressive disease with high morbidity and mortality in which long-term survival rates remain disastrous. Surgical resection is the only potentially curable treatment for early pancreatic cancer; however, the right patient qualification is crucial for optimizing treatment outcomes. With the rapid development of radiographic and surgical techniques, resectability decisions are made by a multidisciplinary team. Upfront surgery (Up-S) can improve the survival of patients with potentially resectable disease with the support of adjuvant therapy (AT). However, early recurrences are quite common due to the often-undetectable micrometastases occurring before surgery. Adopted by international consensus in 2017, the standardization of the definitions of resectable PDAC (R-PDAC) and borderline resectable PDAC (BR-PDAC) disease was necessary to enable accurate interpretation of study results and define which patients could benefit from neoadjuvant therapy (NAT). NAT is expected to improve the resection rate with a negative margin to provide significant local control and eliminate micrometastases to prolong survival. Providing information about optimal sequential multimodal NAT seems to be key for future studies. This article presents a multidisciplinary concept for the therapeutic management of patients with R-PDAC and BR-PDAC based on current knowledge and our own experience.

## 1. Introduction

The incidence of pancreatic ductal adenocarcinoma (PDAC) is increasing worldwide and is associated with high mortality due to its aggressive biology and often-delayed diagnosis [1,2]. According to the American Cancer Society, pancreatic cancer is the third most common cause of cancer-related deaths in the United States [3]. According to the Polish National Cancer Registry 2020, pancreatic cancer is the sixth most common cause of cancer death in men, and the fifth most common cause of cancer death in women, with mortality rates increasing yearly [4]. In 40% of cases, factors are identified that increased the risk of developing pancreatic cancer. Most of them are environmental factors: chronic pancreatitis, alcohol consumption, diabetes mellitus, obesity, tobacco smoking and age. Recent studies have suggested a link between microbiota imbalance and PDAC. Memba et al. showed that lower levels of Neisseria elongate and Streptococcus mitis and higher levels of Granulicatell adiacens are associated with increased risk of PDAC [5]. The incidence rate for both sexes increases with age. PDAC is extremely rarely diagnosed at an early age, and it is usually defined as a disease of elderly people aged over 70 years. Approximately 90% of newly diagnosed patient are over 55 years old, most of them between 70 and 80 years old.

The lack of early symptoms [6] and ineffective screening programs mean that as many as 80% of patients are in an advanced stage of the disease at the time of diagnosis [7]. Surgical resection is the only potentially curative treatment of PDAC; however, no more than 20% of patients have the chance to undergo upfront surgery (Up-S), and less than 30% are considered borderline resectable at the time of diagnosis [8,9]. Adjuvant chemotherapy (AT) has been shown to improve overall survival (OS) and disease-free survival (DFS) postoperatively [10].

Recently, there has been emerging emphasis on neoadjuvant therapy (NAT) as a new therapeutic strategy for early-stage pancreatic cancer to improve the probability of R0 resection [11,12,13] and OS.

Our article presents an approach to treating pancreatic cancer based on a review of key studies focusing on prospective randomized controlled trials. Since more than 90% of pathological diagnoses of pancreatic cancer involve pancreatic ductal adenocarcinoma (PDAC), these terms are used interchangeably throughout this article.

In this review, we describe how we manage resectable and borderline resectable PDAC in an evidence-based manner and based on our clinical experience. The selection of articles for this review was carried out in accordance with the Preferred Reporting Items for Systematic Review and Meta-Analyses (PRISMA) statement. PubMed, Google Scholar and Scopus were systematically searched until February 2023. We reviewed clinical trials and meta-analyses of adjuvant and neoadjuvant PDAC. We searched for studies comparing neoadjuvant chemotherapy with chemotherapy plus preoperative surgery for both R-PDAC and BR-PDAC. The search strategy included multiple combinations of search terms and was limited to English.

## 2. Definition of Resectable Pancreatic Carcinoma (R-PDAC) and Borderline Resectable (BR-PDAC)

**Figure 1:**
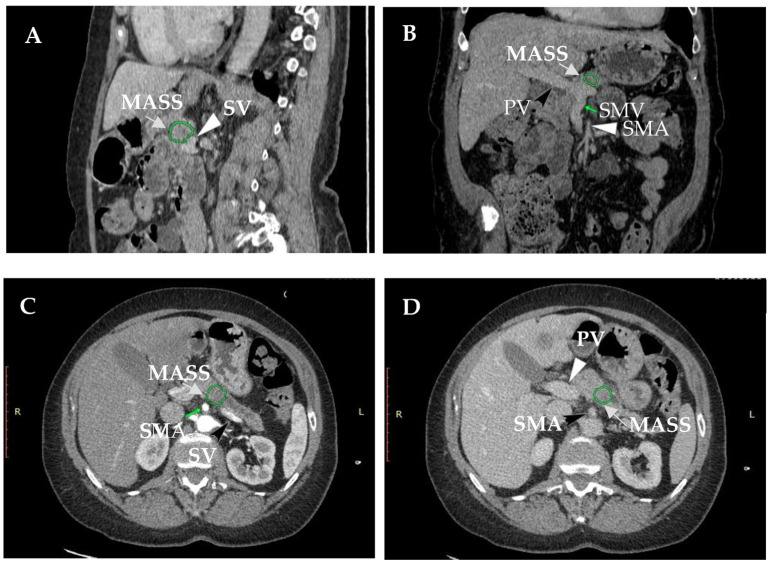
CT image of resectable pancreatic ductal adenocarcinoma (R-PDAC). (**A**,**B**) A coronal and (**C**,**D**) and axial base during the pancreatic phase of CT demonstrating hypovascular mass in pancreatic body without PV, SMA and SV involvement. SMV—superior mesenteric vein, PV—portal vein, SMA—superior mesenteric artery, CA—celiac artery, CHA—common hepatic artery, PHA—proper hepatic artery, SV—splenic vein.

Pancreatic cancer without distant metastases is classified as resectable, borderline or inoperable (locally advanced). Assessment of PDAC resectability is based on preoperative contrast-enhanced multiphase computed tomography (CT) or magnetic resonance imaging (MRI). Generally, a tumor is defined as resectable if it does not infiltrate the major mesenteric blood vessels, i.e., the celiac axis (CA), common hepatic artery (CHA), superior mesenteric artery (SMA), superior mesenteric vein (SMV) and portal vein (PV) [9]. Originally, the resection status was determined based on the probability of obtaining a negative microscopic margin (R0) by assessing the circumferential degree of the tumor contact with the vessels using a CT scan, where <180° contact between tumor and the PV and SMV vessel walls was required (without vein contour irregularities), portal vein/splenic confluence was clear and there was no arterial involvement of the SMA, CA and CHA [13] (Figure 1).

The decision is always made in consultation with a multidisciplinary team that considers not only clinical and radiological characteristics, but also the patient’s psychological approach to surgery and their comorbidities. For some time, it has been recognized that the prognosis for patients undergoing surgical resection for PDAC depends on the margin status. The term “borderline resection” (BR-PDAC) has identified a new group of patients with technically resectable tumors but at high risk of non-radical microscopic resection (i.e., R1) and/or early recurrence after surgery. Histologically, R0 resections are associated with the best outcome, while positive R1 resection margins are associated with reduced survival. Patients with residual tumors (R2 resection) have a similar prognosis as patients without resection [14,15,16]. Hartwig et al. noticed one more dependence; they found that respectability inversely correlated with CA 19-9 levels. The resection rates fall below 70% for levels above 500 IU/mL vs. increase up to 83% for levels between 37 and 100 IU/mL [17]. In turn, Tas et al. reported that PS was the prognostic factor that was the best at predicting OS for all stages of pancreatic cancer. Univariate analysis showed that baseline poor PS of patients (PS 2–4) was significantly associated with shorter survival in localized (*p* = 0.015), locally advanced (*p* = 0.01), metastatic stage (*p* < 0.001) and in the whole group (*p* < 0.001) [18]. The above data indicate the necessity of developing a uniform definition taking into account biological and conditional factors. The International Association of Pancreatology (IAP) in Sendai, Japan, in 2016 defined RB-PDAC as tumor–vein (SMV or PV) contact ≥ 180° with irregular vein outline and/or reconstructable occlusion or tumor–artery (SMA or CA) contact < 180° or reconstructable short segment CHA where the sites of involvement allow safe and complete resection and vessels reconstruction. When assessing resectability, not only the local anatomical conditions are taken into account, but also other factors, such as CA 19-9 antigen level, prediction of regional lymph node metastases based on CT scan, performance status (PS), general condition and comorbidities. Thus, there are three distinct types of BR-PDAC:

(1) BR-type A—evaluates only anatomic features, particularly the relationship between the tumor and peripancreatic vessels;
(2) BR-type B—additionally considers biological factors (CA 19-9 level, lymph nodes) that increase the possibility (but not certainty) of extra-pancreatic disease;
(3) BR-type C—also takes into account some conditional criteria, such as PS and patient comorbidities that may significantly increase the risk of morbidity or mortality after surgery.

Standardization of classifications and ongoing genomic research will contribute to a better understanding of BR-PDAC biology, thereby facilitating the search for optimal treatment options (Table 1) [19].

Anger et al. (2021) retrospectively analyzed 345 patients with BR-PDAC who underwent resection according to ICC. The study compared the cohort of BR-A or BR-B [19] with patients considered primarily resectable (R). Of the patients, 30 were classified as stage BR-A, 62 as stage BR-B and 253 were considered R-PDAC. The median OS in groups was 15 months in group BR-A, 12 months in BR-B vs. 20 months with R-PDAC patients (BR-A vs. R: *p* = 0.09 and BR-B vs. R: *p* < 0.001). It has been demonstrated that optimal preoperative staging, determining surgical resection, is essential for patient survival. Furthermore, BR-B features, in addition to anatomical issues, should be considered when determining eligibility for neoadjuvant treatment [20]. Accordingly, BR-PDAC is usually identified as cancer with aggressive biological behavior, for which a neoadjuvant approach should be preferred over upfront surgery to obtain a radical resection (R0) and avoid early recurrence after surgery [21].

Kato et al. (2019) also supported the validity of the new definitions after retrospectively analyzing 369 patients with PDAC who underwent Up-S. [22]. Based on IAP, patients were classified as R-PDAC (157), BR-PDAC (192) or unresectable UP-RDPC (20), with a median survival time (MST) of 40, 17 and 11 in sequence. Moreover, performance status > = 2 (HR = 2.47, *p* = 0.014) and suspected lymph node in CT (HR = 1.5, *p* = 0.003) were identified as independent prognostic factors by multivariate analysis.

## 3. Adjuvant Treatment (AT)

Adjuvant chemotherapy (AT) has become the standard for managing patients with R-PDAC as it has been demonstrated to improve disease-free (DFS) and overall survival (OS) based on pivotal prospective and randomized clinical trials.

Results of the ESPAC-1 trial showed a significant survival improvement with adjuvant chemotherapy with 5-FU (median OS 19.7 months in the group with chemotherapy vs. 14.0 months without; HR = 0.66, *p* = 0.0005) and no improvement with adjuvant chemoradiotherapy (median OS 15.5 months in the group with chemoradiotherapy vs. 16.1 months without; HR = 1.18, *p* = 0.24) [23].

The CONKO-001 study demonstrated that patients who received adjuvant gemcitabine treatment had a higher chance of disease-free survival compared to those solely under observation (with a median DFS of 13.4 months in the gemcitabine group vs. 6.7 months in the observation group; HR = 0.55, *p* < 0.001). Additionally, patients who received gemcitabine treatment had a prolonged overall survival (with a median OS of 22.8 months compared to 20.2 months in the observation group; HR = 0.76, *p* = 0.03). The study also found that the 5-year overall survival rate was 20.7% in the gemcitabine group compared to 10.4% in the observation group, and the 10-year overall survival rate was 12.2% vs. 7.7% (11).

Final analysis of the ESPAC-3 trial, which compared adjuvant 5-FU with adjuvant gemcitabine, revealed a median survival of 23.0 months for patients treated with 5-FU vs. 23.6 months for those treated with gemcitabine (*p* = 0.39) and median DFS of 14.1 months for 5-FU vs. 14.3 months for gemcitabine (*p* = 0.53), i.e., no significant differences. However, the better tolerability of gemcitabine made the drug the standard of care for adjuvant therapy (ACT) at the time [24].

Another randomized phase III adjuvant trial (ESPAC-4) demonstrated improved overall survival of patients treated with a combination of gemcitabine and capecitabine compared to patients treated with single-agent gemcitabine: 28.0 vs. 25.5 months (*p* = 0.032) [25]. Finally, the breakthrough study in the world of resectable pancreatic cancer was the PRODIGE 24/CCTG PA.6 trial with a modified FOLFIRINOX regimen (fluorouracil, irinotecan, leucovorin, oxaliplatin), which showed a median overall survival of 54.4 months compared to 35 months for gemcitabine used in monotherapy (HR = 0.64, *p*  =  0.003) and established the standard in adjuvant management [26,27] in R-PDAC. A phase II randomized clinical trial—SWOG-S1505—evaluated the effectiveness of perioperative chemotherapy (modified FOLFIRINOX vs. gemcitabine plus nab-paclitaxel—GCB/nabPXL) in a group of 102 patients with resectable PDAC. The two-year OS was 47% for the mFOLFIRINOX arm and 48% for the GCB/nabPXL arm. The median OS was 23.2 months and 23.6 months, respectively. However, neither arm exceeded a statistically significant difference for the 2-year survival threshold, estimated a priori at 40% [28].

The role of chemoradiotherapy in adjuvant therapy is unclear. ESPAC-1 and EORTC trial 40,891 [29] did not show greater survival benefits for those treated with CRT compared with for those under observation. On the other hand, in the retrospective analysis based on the SEER (Surveillance, Epidemiology and End Results) database, adjuvant CRT was associated with improved survival compared with adjuvant chemotherapy in particular subgroups of patients. The benefit was more significant in patients who were females (HR = 0.860; *p* = 0.005) and had pT3 (HR = 0.905; *p* = 0.04) and positive lymph nodes (HR = 0.88; *p* = 0.005) [30].

Another issue currently awaiting clarification is the role of AT in patients with BR-PDAC who have received NAT. The ASCO guidelines recommends AT treatment for 6 months, including the preoperative period, and NCCN also suggests taking it into account. According to a retrospective review by Ivey et al. [31], postoperative chemotherapy after NAT and resection was associated with improved OS (28.7 vs. 20.4 months, *p* = 0.006) for patients with lymph node metastasis. Among node-positive patients, postoperative chemotherapy was associated with improved median OS (27.2 vs. 10.5 months, *p* = 0.001). Among node-negative patients, postoperative chemotherapy was not associated with a survival benefit (median OS, 30.9 vs. 36.9 months, *p* = 0.406). The above data indicate a significant benefit from postoperative chemotherapy in patients after NAT with affected lymph nodes after surgery.

## 4. Neoadjuvant Treatment (NAT)

Unfortunately, many patients who can undergo a radical resection ultimately develop disease recurrence, most often identified as metastatic disease. This suggests that even patients with radiologically localized disease may already have micrometastases, and visible metastases may occur soon after surgery [32]. Therefore, important questions arise as to whether neoadjuvant therapy should be used in all patients eligible for surgery or in specific groups and what kind of NAT should be preferable [33,34].

The first prospective randomized phase II/III trial in South Korea showed the benefits of neoadjuvant treatment in BR-PDAC compared to Up-S. The group treated with gemcitabine-based chemoradiotherapy had a median survival of 21 months vs. 12 months for the upfront surgery group (*p* = 0.02 and R0 resection rate of 51.8% vs. 26.1% (*p* = 0.004)) [12]. The next randomized trial—Prep-02/JSAP-05, comparing gemcitabine and S1 (NAT-GS) with Up-S—showed a median OS of 36.7 months in the NAC-GS group vs. 26.6 months in the Up-S group; HR = 0.72 (*p* = 0.015) [35].

The first large prospective, randomized and multicenter trial—PREOPANC—compared preoperative chemoradiotherapy and upfront surgery for patients with R-PDAC—defined as no arterial involvement and less than 90° venous involvement and RB-PDAC. The primary objective of the study was to increase median survival from 11 to 17 months. The patients were assigned randomly to either receive preoperative chemoradiotherapy or to undergo immediate surgery. The former involved a cycle of gemcitabine (1000 mg/m^2^ on days 1 and 8) followed by radiotherapy (36 Gy, 15 fr) with typical administration of gemcitabine, another cycle of gemcitabine, surgery and 4 months of adjuvant gemcitabine. The latter involved immediate surgery followed by 6 months of adjuvant gemcitabine. Median overall survival by intention-to-treat analysis was 16.0 months with preoperative chemoradiotherapy vs. 14.3 months with immediate surgery (HR = 0.78; *p* = 0.096). The resection R0 rate was 71% for preoperative chemoradiotherapy vs. 40% for patients assigned to immediate surgery (*p* < 0.001). Preoperative chemoradiotherapy was associated with significantly better disease-free survival (*p* = 0.032) and locoregional failure-free interval (*p* = 0.0034) but not with the distant metastasis-free interval (*p* = 0.240). The final analysis showed improved median survival with preoperative chemoradiotherapy (35.2 vs. 19.8 months, *p* = 0.029) for patients who underwent tumor resection and started adjuvant chemotherapy [36].

Cloyd et al. retrospectively analyzed patients with R-PDAC who were treated with chemotherapy or CRT before operation [37]. The chemoradiotherapy group was associated with R0 resection (91% vs. 79%, *p* < 0.01), being nodenegative (53% vs. 23%, *p* < 0.01), less locoregional recurrence and better median OS (33.6 vs. 26.4 months, *p* = 0.09). Similar observations were published by Cloyd et al. in a meta-analysis of six randomized clinical trials comparing NAT to Up-S for R-PDAC and BR-PDAC. They proved that NAT significantly improved OS in both groups. Four trials used neoadjuvant CHRT, and two used systemic gemcitabine-based chemotherapy. Median OS across all studies was higher among patients who received NAT than among those who received Up-S (25.4 months vs. 19.4 months, *p* < 0.001). In subset analyses, the pooled HR remained significantly in favor of NAT, independent of anatomic classification (R-PDAC: HR = 0.73, 95% CI = 0.59–0.91; BR-PDAC: HR = 0.51, 95% CI = 0.28–0.93) or NAT type (CHRT: HR = 0.77, 95% CI = 0.61–0.98; chemotherapy alone: HR = 0.68, 95% CI = 0.54–0.87). Moreover, NAT increased the likelihood of an R0 resection and increased the rate of pathology-negative lymph nodes [38].

A meta-analysis of seven randomized controlled trials also confirmed the benefits of NAT in BR-PDAC. All trials included a neoadjuvant gemcitabine-based chemo(radio)therapy arm. Results showed improved OS from 19 to 29 months compared with that of upfront surgery (HR = 0.66, *p* = 0.001). Further analysis implied that the difference was significant only in the subgroup of BR-PDAC (venous contact > 180°, any arterial contact) (HR = 0.61, *p* = 0.004). In the subgroup of R-PDAC, there was no statistically significant difference in OS (HR = 0.77, *p* = 0.18) [39] (Table 2).

Another meta-analysis of randomized trials showed the benefit of NAT compared with Up-S in R-PDAC. The meta-analysis encompassed six trials in which 469 patients were assigned to NAT (*n* = 212) or Up-S (*n* = 257) groups. Compared with Up-S, NAT significantly improved both OS (HR = 0.75; *p* = 0.033) and DFS (HR = 0.73; *p* = 0.002). Moreover, the R0 resection rate was substantially higher for NAT than for Up-S (RR = 1.31; *p* = 0.0004) [40]. Receiving NAT is also a significant advantage for patients at risk of postoperative complications [41], which can delay the administration of AT [14,42].

Considering the results following FOLFIRINOX-based AT, it was necessary to determine whether this regimen would also work in preoperative treatment. The results of the first trials of NEPAFOX [43] and NEONAX [44] did not confirm the intended goals due to low numbers of randomized patients. They did not reach the primary endpoint, DFS at 18 months, in the modified intention-to-treat (ITT) population.

Supporting evidence came from the ECPAC-5F trial of Up-S vs. NAT chemotherapy CHRT. Patients with BR-PDAC were randomly assigned to one of three arms: (1) Up-S; (2) NAT GemCap (gemcitabine, capecitabine); (3) FOLFIRINOX or CRTH (50.45 Gy in 28 daily fractions with capecitabine throughout). All patients received AT after surgery. The primary endpoint R0/R1 resection rate was comparable for Up-S (44%) and NAT (41%; *p* = 0.668). The secondary outcome revealed a significant benefit in terms of OS at 1 year with 12-month survival rates of 77% in the NAT group compared to 42% in the Up-S group (HR = 0.28; *p* < 0.001). The highest survival rate of 84% was achieved in the FOLFIRINOX group (vs. 79% with GEMCAP vs. 64% with CHRT vs. 42% with Up-S) [45,46].

Janssen et al. compared outcomes from 15 studies (512 patients with R-PDAC and BR-PDAC) investigating the efficacy of FOLFIRINOX alone vs. FOLFIRINOX and radiotherapy. The pooled estimated median OS was 21.6 months for FOLFIRINOX alone vs. 22.4 months for FOLFIRINOX with radiotherapy. The pooled R0 resection rate was higher for FOLFIRINOX with radiotherapy (97.6% vs. 88.0%, *p* = 0.045) [47].

Murphy et al. conducted a single-arm phase II clinical trial on patients with BR-PDAC. They enrolled 48 of 50 patients who received chemotherapy 8xFOLFIRINOX, and, after restaging, those with resolution of vascular involvement underwent short-course chemoradiotherapy (5Gyx5 with protons) and capecitabine. In cases with persistent vascular involvement, patients underwent long-course chemotherapy with capecitabine or 5FU. Surgery was performed in 81% of cases with resection of 66% patients. R0 resection was achieved in 97% of resectable tumors. Median OS was 37.7 months among eligible patients; in the group that underwent resection, the median OS was not reached [48].

Katz et al. showed in a phase II clinical trial—Alliance A021501—that NAT with mFOLFIRINOX alone was associated with favorable OS in patients with BR-PDAC compared to mFOLFIRINOX in combination with hypofractionated radiotherapy [49]. One hundred and twenty-six patients were enrolled to two groups. Arm 1 received eight cycles of mFOLFIRINOX; arm 2, following completion of seven cycles of mFOLFIRINOX, received stereotactic irradiation (SBRT) (33–40 Gy in 5 fr) or image-guided radiotherapy (IGRT) (25 Gy in 5 fr) before surgery. Then, patients were restaged with CT or MRI and underwent surgery within 1–2 months. Postoperatively, four cycles of FOLFOX6 were administered in both arms. An initial analysis of the 30 enrolled patients in each arm showed surprising results. R0 resection was achieved in 57% of arm 1 vs. 33% in arm 2. Therefore, further recruitment in arm 2 was terminated. Similar disturbing results were obtained for the median OS (29.8 vs. 17.1). Finally, pancreatectomy was performed for 49% in arm 1 and 35% in arm 2 with R0 resection in 88% vs. 74% accordingly. Unfortunately, the results in the radiotherapy arm were significantly worse compared to those in other studies.

More support for CHRT came from the Alliance A021101 trial of NAT with mFOLFIRINOX followed by capecitabine-based chemoradiation. In that study, 68% of patients ultimately underwent pancreatectomy, 93% of whom achieved microscopically negative margins [50].

## 5. Discussion

Pancreatic cancer, even when resectable (R-PDAC) or borderline resectable (BR-PDAC), must be presumed to be a systemic disease that benefits from multimodal treatment.

According to the literature and our clinical experience, Up-S followed by adjuvant treatment remains the standard of care in R-PDAC. However, in assigning our patients with R-PDAC to Up-S, we consider multiple prognostic aspects that reflect the aggressiveness of the disease and likelihood of occult metastases. Very high CA 19-9 levels (>500 U/mL) and suspicious radiologic findings (e.g., ≤10 mm lymph nodes in CT scan) should warrant consideration of neoadjuvant therapy. A chemotherapy regimen of 12xmFOLFIRINOX is, according to literature and our experience, the gold standard for a patient with a good performance status. In case of frail or elderly patients with comorbidities, we consider a different chemotherapy regimen: gemcitabine +/− capecitabine also for 6 months. In cases of microscopically incomplete resection (R1) and with evidence of lymph node metastasis in histopathological examination post surgery, we consider capecitabine-based chemoradiotherapy after adjuvant CTH.

In treatment of BR-PDAC, we follow the guidelines of neoadjuvant therapy which are recommended by the American Society of Clinical Oncology (ASCO), the National Comprehensive Cancer Network (NCCN) and the American Society for Radiation Oncology (ASTRO). In our opinion, the mFOLFIRINOX chemotherapy regimen is the standard of care for most patients. For frail patients, we use gemcitabine plus capecitabine. We do not apply a gemcitabine/nab-paclitaxel regimen due to the lack of reimbursement for neoadjuvant therapy in Poland. CRTH is administered to specific patients who have not responded partially to neoadjuvant chemotherapy before undergoing surgical resection. Our recommendations for adjuvant treatment are the same as for R-PDAC.

**Figure 2:**
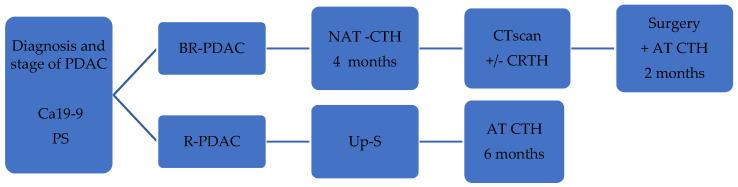
Proposed treatment algorithm. Diagnosis and stage of PDAC confirmed by CT/MRI. CA 19-9 more than 500 IU/mL, suspicious regional lymph metastasis in CT scan, PS = 2—consideration for NAT. Abbreviations: PDAC—pancreatic ductal adenocarcinoma, CA 19-9—carbohydrate antigen 19-9, PS—performance status, BR-PDAC—borderline resectable PDAC, R-PDAC—resectable PDAC, NAT-CTH—neoadjuvant chemotherapy, Up-S—upfront surgery, CT—computed tomography, CRTH—chemoradiotherapy, AT CTH—adjuvant chemotherapy.

Drawing on published studies, guidelines and our expertise, we have created a diagram outlining a proposed approach for treating R-PDAC and BR-PDAC (Figure 2).

The role of adjuvant treatment (AT) and neoadjuvant treatment (NAT) for R-PDAC and BR-PDAC pancreatic cancer is undergoing continuous evolution.

A prospective multicenter controlled, phase II study, PANACHE01-PRODIGE 48, confirmed the feasibility and efficacy of NAT mFOLFIRINOX in patients with R-PDAC [51]. These results support the ongoing clinical trial PREOPANC-3 [52], which is comparing perioperative versus adjuvant FOLFIRINOX for PDAC. The ongoing Alliance A021806 [53] trial of perioperative vs. adjuvant chemotherapy for R-PDAC cancer may set new treatment standards. Patients will be randomized to treatment with NAT with mFOLFIRINOX (perioperative arm) or Up-S, followed by AT-mFOLFIRINOX (adjuvant arm). A subsequent PREOPANC-3 trial will compare perioperative versus adjuvant FOLFIRINOX for PDAC. Patients with R-PDAC will be randomly assigned (1:1) to eight cycles of neoadjuvant mFOLFIRINOX followed by surgery and four cycles of adjuvant mFOLFIRINOX (arm 1) or Up-S followed by twelve cycles of adjuvant mFOLFIRINOX (arm 2). Both studies are expected to clarify the role and place of neoadjuvant treatment in R-PDAC. The final results of the PREOPANC-2 study [54], which directly compared two neoadjuvant approaches (eight cycles of FOLFIRINOX followed by surgery and without adjuvant therapy vs. three cycles of gemcitabine with hypofractionated radiotherapy during the second cycle followed by surgery and four cycles of adjuvant gemcitabine) in patients with R-DAC and BR-PDAC, should provide an answer to the question about the role of chemoradiotherapy in neoadjuvant treatment. PANDAS-PRODIGE 44 (NCT02676349) is another ongoing randomized phase II trial designed to compare NAT with mFOLFIRINOX with or without preoperative CRTH in patients with BR-PDAC.

Immunotherapy still has not yet achieved significant success in treating pancreatic cancer. Researchers continue to explore different strategies and combinations of therapies to improve outcomes. A randomized trial of patients with BR-PDAC treated with chemotherapy with or without algenpantucel-L immunotherapy Did not show improved survival [55]. There is an ongoing study, NCT 03983057, on the therapeutic effect of the combination of anti-PD-1 antibody with mFOLFIRINOX vs. mFOLFIRINOX alone in patients with BR-PDAC and locally advanced pancreatic cancer.

Another aspect that requires more research is the diverse molecular profile of the tumor, which contributes to the poor prognosis for those with PDAC. More than 95% of pancreatic cancer cases have genetic changes [56]. Reddy et al. [57] observed in their retrospective analysis that groups of 35 patients treated with mFOLFIRINOX chemotherapy, gemcitabine and nab-paclitaxel or gemcitabine and capecitabine followed by SBRT with KRAS G12V and CDKN2A showed a better rate of pathological tumor regression in NAT than patients treated with others. Similarly, NOTCH1/2 mutations were associated with poorer OS, PFS and distant metastasis-free survival. Reyes pointed out that, from the point of view of the surgeon, the molecular subtypes of PDAC are currently irrelevant in everyday clinical practice. However, it is possible that a specific molecular profile influences the delay in response to treatment and thus the need for resection before or after surgery [58].

There is also an urgent need for a clear definition of oligometastatic status in the context of pancreatic cancer. It is crucial for consistent classification and management of this group of patients. Traditionally, surgery has not been recommended for advanced PDAC with distant metastases. However, recent advancements in treatment have led to the consideration of surgery even in metastasic disease. Tachezy and Gebauer [59] reported a longer median OS (14.5 months vs. 7.5 months) in patients undergoing hepatic metastasectomy vs. in those undergoing palliative bypass surgery. Kandel et al. [60] noticed a significantly longer OS (32.4 months in the M1 surgery group vs. 11.7 months in the M1 no-surgery group). Damanakis et al. [61] proposed a definition of oligometastatic disease including anatomical and biological criteria such as: ECOG 0/1, ≤4 hepatic or pulmonary lesions, no ascites, liver cirrhosis or lung emphysema, <1000 U/mL CA 19-9 and response to chemotherapy or stable disease and resectability of primary tumor. One of the challenges in the treatment of oligometastatic pancreatic cancer is the identification of appropriate biomarkers that can predict treatment success and guide decisions regarding surgical interventions.

Targeting autophagy is also another exciting field for future research. In a study by Yang et al. [62], it was shown that the pathogenesis of pancreatic cancer has a distinct dependence on autophagy—the pathway of cellular organelle degradation regulates chemotherapy sensitivity in pancreatic cancer. Pharmacologic inhibition of autophagy by chloroquine leads to increased reactive oxygen species, DNA damage and metabolic defect, leading to decreased mitochondrial oxidative phosphorylation and thus regression of pancreatic tumors. Similarly, the inhibition of autophagy by deficiency of the labile iron pool is responsible for the reduction of mitochondrial respiration [63]. This supports the critical link between autophagy, iron metabolism and mitochondrial function, which may have implications for PDAC progression. Additionally, Chen et al. constructed an autophagy-related mRNA/miRNA/TF/immune cells network based on the best-in-class algorithms and multiomics analysis and tested the drug sensitivity to detect a potential signal pathway which might be a possible target of autophagy modulators [64]. However, as with any research, further studies are needed to validate these findings and determine the clinical applicability of modulating autophagy in PDAC treatment.

## 6. Conclusions

This article, a comprehensive review based on the current literature and our knowledge, summarizes the current medical knowledge on management strategies for resectable and potentially resectable pancreatic cancer.

PDAC, due to low survival rates, requires a constant search for better imaging methods for faster diagnosis and optimal staging before the decision regarding the best method of treatment is made.

The new definition of PDAC that has been developed, taking into account anatomical, biological and conditional factors, seems to be a prelude to further improvements. The therapeutic sequences of neoadjuvant treatment–resection for BR-PDAC and upfront resection–adjuvant treatment for R-PDAC seem to be becoming standard, which was shown in this review. There is still uncertainty about the additional benefits of radiotherapy added to chemotherapy and the sequence of such treatment, as we have highlighted in this article on the treatment of R-PDAC and BR-PDAC. Further randomized trials are needed to determine the optimal management. A better understanding of the molecular basis of PDAC seems to also be important, as it will undoubtedly have an impact on the development of optimal management.

## Figures and Tables

**Table 1:** International definitions of R-PDAC and BR-PDAC based on anatomical, biological and conditional aspects.

Type of Definition	Anatomical	Biological	Conditional
		No: R-Type A	No: R-Type A
R-PDAC	R-Type A		
		Yes: BR-Type B	Yes: BR-Type C
			
		No: BR-Type A	No: BR-Type A
BR-PDAC	BR-Type A		
		Yes: BR-Type AB	Yes: BR-Type AC

Anatomical definition: R-Type A: SMV/PV: no tumor contacts or unilateral. SMA, CA, CHA: no tumor contact; BR-Type A: SMV/PV: tumor contact ≥ 180° or bilateral narrowing/occlusion, not exceeding inferior border of duodenum. SMA, CA: tumor contact < 180° without deformity/stenosis. CHA: tumor contact, without contact with PHA and/or CA. Biological definition: CA 19-9 more than 500 IU/mL, regional lymph metastasis (biopsy or PET-CT). Conditional definitions: depressed performance status (PS = 2 or more).

**Table 2:** Neoadjuvant radio/chemoradiotherapy in pancreatic cancer.

Study	Evidence	Protocol	RT Protocol	% R0	% N0	Results
PREOPANC1	prospective	R-PDAC, BR-PDAC: Up-S–6xGem vs. 3xGem–3xGem/RT–Up-S–4xGem	GTV + ILN 15 × 2.5 Gy	40% vs. 71%	23% vs. 53%	Median OS: 14.3 m vs. 15.7 m, DFS: 7.7 m vs. 8.1 m
Cloyd	retrospective	R-PDAC: 4–6xGem/5FU-based regimen–S vs. Gem or Cap + RT–S	3DCRT: GTV + 10 mm + ENI, 10 × 3 Gy or 28 × 1.8 Gy	79% vs. 91%	22% vs. 53%	Median OS: 26.4 m vs. 33.6 m
A021501	prospective	BR-PDAC: 8xFOLFIRINOX–S–4xFOLFOX6 vs. 7xFOLFIRINOX–RT–S–4xFOLFOX6	SBRT: GTV + 3 mm (5 × 6.6–8 Gy) or IMRT: GTV + 5–10 mm (5 × 5 Gy)	88% vs. 74%	47% vs. 47%	Median OS: 29.8 m vs. 17.1 m DFS 15.0 m vs. 10.2 m
Jannsen	prospective	RPDAC, BR-PDAC: FOLFIRINOX–S vs. FOLFIRINOX + RT-S	GTV + ILN or SBRT (25–50.4 Gy)	88% vs. 97%	52% vs. 67%	Median OS: 21.6 m vs. 22.4 m
ESPAC-5	prospective	B-PDAC: Up-S vs. NAT-S, GemCap or FOLFIRINOX Cap + RT AT: Gem or GemCap or mFOLFIRINOX	GTV + ILN (28 × 1.8 Gy)	14% vs. 30% (18% vs. 18% vs. 37%)		1-year DFS: 33% vs. 59%
Murphy	prospective	B-PDAC: 8xFOLFIRINOX.—Cap + RT—S	GTV + 1 cm ENI short course: Protons 5 × 5 Gy or Photons 10 × 3 Gy or Long course: 28 × 1.8 Gy	97%		Median OS: 37.7 m 2-years OS: 56%

Gem—gemcitabine, RT—radiotherapy, Up-S—upfront resection, GTV—gross tumor volume, ENI—elective nodal irradiation, ILN—involved lymph nodes, SBRT—stereotactic body radiotherapy, IMRT—image-guided radiotherapy, OS—overall survival, 5FU—5-fluorouracil, Cap—capecitabine.

## Abbreviations

PDAC	pancreatic ductal adenocarcinoma
Up-S	upfront surgery
AT	adjuvant therapy
R-PDAC	resectable pancreatic ductal adenocarcinoma
BR-PDAC	borderline pancreatic ductal adenocarcinoma
NAT	neoadjuvant therapy
OS	overall survival
DFS	disease-free survival
CT	computed tomography
MRI	magnetic resonance imaging
CA	celiac axis
CHA	common hepatic artery
SMA	superior mesenteric artery
SMV	superior mesenteric vein
PV	portal vein
NCCN	National Comprehensive Cancer Network
MDACC	Anderson Cancer Center
AHPBA	American Hepato-Pancreato-Biliary Association
SSO	Society of Surgical Oncology
SSAT	Society for Surgery of the Alimentary Track
PS	performance status
IAP	International Association of Pancreatology
CRTH	chemoradiotherapy
ASCO	American Society of Clinical Oncology
ASTRO	American Society for Radiation Oncology
AJCC	American Joint Committee on Cancer
mFOLFIRINOX—a chemotherapy regimen: FOL—folinic acid, F—fluorouracil (5-FU), IRI—irinotecan, OX—oxaliplatin	
